# MAFLD and Celiac Disease in Children

**DOI:** 10.3390/ijms24021764

**Published:** 2023-01-16

**Authors:** Serena Scapaticci, Annamaria Venanzi, Francesco Chiarelli, Cosimo Giannini

**Affiliations:** Department of Pediatrics, University of Chieti ‘G. d’Annunzio’, 66100 Chieti, Italy

**Keywords:** celiac disease, pediatric non-alcoholic fatty liver disease, metabolic associated fatty liver disease, children

## Abstract

Celiac disease (CD) is an immune-mediated systemic disorder elicited by the ingestion of gluten whose clinical presentation ranges from the asymptomatic form to clinical patterns characterized by multiple systemic involvement. Although CD is a disease more frequently diagnosed in patients with symptoms of malabsorption such as diarrhea, steatorrhea, weight loss, or failure to thrive, the raised rate of overweight and obesity among general pediatric and adult populations has increased the possibility to diagnose celiac disease in obese patients as well. Consequently, it is not difficult to also find obesity-related disorders in patients with CD, including “metabolic associated fatty liver disease” (MAFLD). The exact mechanisms linking these two conditions are not yet known. The going assumption is that a gluten-free diet (GFD) plays a pivotal role in determining an altered metabolic profile because of the elevated content of sugars, proteins, saturated fats, and complex carbohydrates, and the higher glycemic index of gluten-free products than gluten-contained foods, predisposing individuals to the development of insulin resistance. However, recent evidence supports the hypothesis that alterations in one of the components of the so-called “gut–liver axis” might contribute to the increased afflux of toxic substances to the liver triggering the liver fat accumulation and to the subsequent hepatocellular damage. The aim of this paper was to describe the actual knowledge about the factors implicated in the pathogenesis of hepatic steatosis in pediatric patients with CD. The presented review allows us to conclude that the serological evaluations for CD with anti-transglutaminase antibodies, should be a part of the general workup in the asymptomatic patients with “non-alcoholic fatty liver disease” (NAFLD) when metabolic risk factors are not evident, and in the patients with steatohepatitis when other causes of liver disease are excluded.

## 1. Introduction

CD is an immune-mediated systemic disorder triggered by the consumption of gluten proteins in genetically susceptible individuals. The disease is characterized by a variable combination of gluten-related signs and symptoms, CD-specific antibodies, HLA-DQ2 or HLA-DQ8 haplotypes, and enteropathy [1].

It is the most common immune-mediated enteropathy in Western countries [2] whose spread has progressively increased in recent years. Although one of the reasons of its prevalence partly lies in the improvement in diagnostic tools, the screening programs of populations at risk of developing CD have certainly contributed to the precocious detection of individuals affected. Currently, the reported prevalence of CD worldwide is around 1% in the general population with an estimated rate ranging from 1:80 to 1:300 in European, American, and Australian children (3 to 13 per 1000 children) [3]. Although the rate of diagnosis is increasing, it is believed that most patients with CD go undiagnosed [4]. The diagnostic difficulties are partly due to the wide spectrum of the possible manifestations of the disease. In recent years, there has been a shift in the clinical presentation of CD from the classical form to the non-classical, oligosymptomatic, and asymptomatic forms [3,5]. Riznik et al. [3] carried out a study enrolling 653 children and adolescents with CD from different European countries showing that the age at diagnosis of CD has increased during the past decades with an actual median age of 7 years. Furthermore, the authors reported that the classic CD triad of symptoms (diarrhea, distended abdomen, abdominal pain) is more frequent in younger children, while with increasing age, atypical symptoms become more common. Although CD is a disease more frequently diagnosed in patients with symptoms of malabsorption such as diarrhea, steatorrhea, weight loss, or failure to thrive [4], the raised rate of overweight and obesity among general pediatric and adult populations has increased the possibility to diagnose CD in obese patients as well. In addition, several studies have supposed that a gluten-free diet (GFD) might predispose individuals to adipose tissue accumulation thus inducing an increased prevalence of overweight and obese patients with CD [6,7]. Therefore, it is not difficult to also find obesity-related complications in CD patients. Particularly, a non-casual coexistence of CD and NAFLD has been reported both in adult and pediatric patients [4]. Beyond a genetic predisposition, this group of diseases is a consequence of a general metabolic dysregulation which have been recently described in a new definition, namely the “metabolic associated fatty liver disease” (MAFLD) [8,9]. The pathogenetic link between CD and NAFLD/MAFLD is not yet known. Whilst a GFD may contribute to an unhealthy metabolic profile, recent studies have pointed out that CD and its intestinal alterations could cause an altered exposition of the liver to a substance able to enhance hepatic damage [10]. The aim of this review is to describe the pathogenic link between CD and MAFLD, analyzing the so-called gut–liver axis and the possible pathological implications of this association.

## 2. Research Strategy

We proceed with a review of data presented in the literature on the relationship between CD and hepatic steatosis, with a major focus on obese children and adolescents, through one of the main providers of academic search engines (PubMed). We analyzed articles and guidelines of major international scientific societies of the last 30 years using keywords including “celiac disease”; “pediatric non-alcoholic fatty liver disease”; “metabolic associated fatty liver disease”; “children”.

## 3. CD and MAFLD

NAFLD is a chronic hepatic disease not secondary to genetic/metabolic disorders, infections, use of medications, ethanol consumption, or malnutrition [11]. Currently, it is one of the most common forms of chronic liver disease in the general population worldwide, whose prevalence is mainly and positively correlated to the increased rate of obesity [12,13,14,15]. Nowadays, NAFLD is a major cause of chronic hepatic involvement. It is reported that the NAFLD global prevalence is equal to 34% among overweight or obese pediatric subjects between the ages 1–19 years old and 45% in those settings based on child obesity clinics [16]. Different adult studies have observed that CD and hepatic steatosis are diseases highly associated with each other. Particularly, the prevalence rate of CD in patients with NAFLD is reported to be 2–14% [17]. Furthermore, in patients with CD, the risk of NAFLD development is between 4 and 6 times higher than the general population [18,19]. Data about the association of pediatric CD and NAFLD are inconsistent. In a pediatric study evaluating 120 children with obesity undergoing evaluation for NAFLD, none of subjects studied presented a positivity in CD serology [20]. In a recent large multicentric study involving obese and overweight children aged 10–16 years evaluating for aminotransferase elevation or hepatic steatosis, the authors diagnosed CD only in 3 out of the 900 patients enrolled, confirming the prevalence reported in the general population [21]. However, IgA deficiency, classically associated with CD [20], has been identified in 5% of patients with NAFLD supposing an increasing risk of CD developing [21]. The highest incidence of the NAFLD diagnosis has been reported in the first five years of CD diagnosis but it remains documentable until 15 years after [19]. This epidemiological trend might partially be attributable to dietetic imbalances derived from the starting of a GFD that bring an increased intake of lipids and cholesterol and a decreased intake of fiber to the diet [17,19]. As a consequence, patients with CD develop an impaired metabolic profile raising the risk of Metabolic Syndrome (MetS) and consequently MAFLD. Therefore, MAFLD diagnosis requires the presence of hepatic steatosis by using histological , imaging (ultrasound), or blood biomarker (e.g., ALT) tests, combined with altered corporeal fat accumulation/distribution, alterations in glucose homeostasis (prediabetes or type 2 Diabetes Mellitus “T2DM”), or evidence of MetS [9]. The consensus about the definition of MetS in children is not univocal. The International Diabetes Federation (IDF) has proposed to diagnose MetS in children in the presence of two or more altered biometric parameters, including waist circumference, blood pressure, triglycerides (TGs), high density lipoprotein–cholesterol (HDL-C) levels, fasting glucose, and TG</HDL-C ratio, standardized for age, sex, and ethnicity [22].

Nonetheless, the loss of gut integrity combined with alterations in gut microbiome and other components of the gut–liver axis might play a role in the intricate pathophysiological pathway of NAFLD/MAFLD development [23]. Finally, recent reports suggest that CD may occur as a consequence of malnutrition secondary to intestinal malabsorption [24].

**Figure 1:**
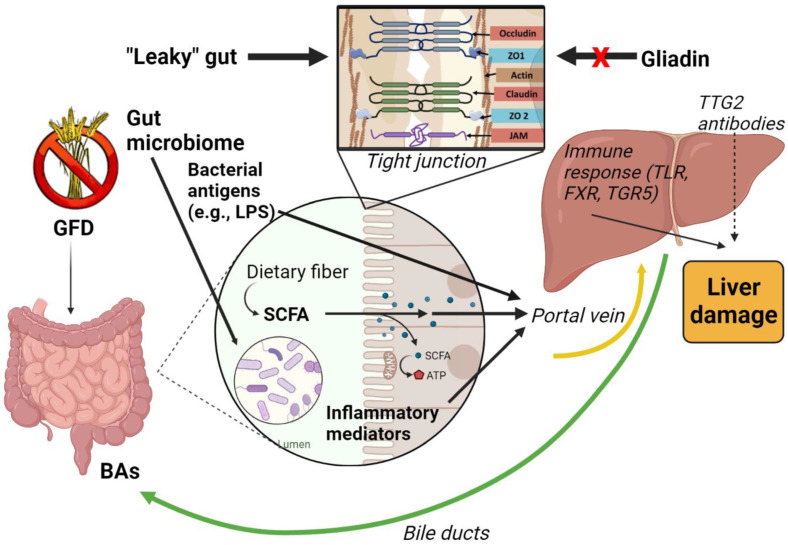
The homeostasis of the intestinal–liver axis is ensured by a healthy intestinal barrier and a balanced and functional microbiome, which act in parallel by preventing elevated levels of toxic substances from reaching the liver through the portal circulation. Increased permeability, dysbiosis, and the production of toxic metabolites may combine to determine a pathological gut–liver axis that contributes to the pathogenesis of NAFLD. Created with BioRender.com (accessed on 13 January 2023).

In the following paragraphs, we will analyze how these aforementioned mechanisms contribute to NAFLD/MAFLD development in CD patients (Figure 1).

## 4. The Gut–Liver Axis

The so-called “gut–liver axis” has gained a progressive interest in the last few years. It is a dynamic system composed of various components (intestinal barrier, gut microbiota, bile, lymphocytes and hepatic receptors likely PRRs, farnesoid X receptor (FXR), Takeda G-protein receptor 5 (TGR5), fibroblast growth factor receptor 4 (FGFR4)), which are involved in mediating metabolic, motor, neuroendocrine, and immunological functions [10]. Gut and liver have a bidirectional interaction, characterized by a close anatomical and functional link, mainly due to the portal circulation, which is responsible for carrying gastrointestinal luminal contents, such as dietary nutrients but also toxins, xenobiotics, and bacterial metabolites, to the liver. In turn, the liver produces signals to the gut by secreting bile and microbicidal substances in the bile ducts able to interfere with the gut functionality. An impaired functionality of the gut–liver axis leads to an unregulated hepatic immunological response which may play a role in the appearance and progression of liver damage. The intestinal mucous membranes and vascular vessels are the main barrier components of the gut–liver axis which prevent dangerous hits from the gut in reaching the portal circulation in healthy conditions. The disruption of the intestinal barrier, due to increased intestinal permeability or altered intestinal immunity, increases the afflux of molecules able to activate gut-associated lymphatic tissue (GALT) (e.g., lipopolysaccharide (LPS), peptidoglycans, super-antigens, bacterial DNA, flagellin, and heat shock proteins). Consequently, intestinal levels of pro-inflammatory cytokines (TNF, IL-6, IL-1), chemokines, and eicosanoids increase. Unfortunately, GALT itself does not entirely remove the antigens to which it is exposed. Therefore, together with intestinal inflammatory cells, pro-inflammatory molecules, and bacterial metabolites (such as ethanol, acetaldehyde, trimethylamine, short chain fatty acids (SCFAs), and free fatty acids, food and bacterial antigens), they reach the liver where there is a local activation of the immune system promoting liver damage. The relationship existing between the alteration in the gut barrier and hepatic inflammation has been confirmed by the evidence that both increase intestinal permeability and high levels of proinflammatory cytokines and chemokines characterize people with hepatic damage. In addition, elevated levels of bacterial LPS have been found in the portal and/or systemic circulation (endotoxemia) in patients with chronic liver diseases [25,26,27,28]. Therefore, the increased permeability of the intestinal barrier in CD patients alters the gut–liver axis and contributes to the pathogenesis of hepatic disorders, such as MAFLD.

A pivotal role is also played by the gut microbiota which comprises trillions of microorganisms such as bacteria, viruses, archaea, eukaryotes, protozoa, and their collective genome (named “microbiome”) that establish a symbiotic relationship with the host. The microbiota modulates the gut barrier integrity and takes part in digestion, promotion of angiogenesis, and nerve function [29]. Thanks to the release of metabolites, peptides, and hormones capable of activating the immune system, the microbiota also shapes host immunity and metabolism [30,31] and has an impact on the development of cardiovascular, endocrine, gastrointestinal, and hepatic diseases, arising as a “new virtual metabolic organ” [32]. Therefore, it is clear that the homeostasis of the gut microbiome and the integrity of the intestinal barrier protect liver cells from excessive exposure to microorganism components, bacterial products, fatty acids, or modified bile composition [33]. The bacterial products and metabolites which manage to pass the intestinal barrier and reach the liver can contribute to the pathogenesis of liver diseases, triggering an inflammatory response and the release of cytokines [34,35]. Therefore, the increased permeability of the intestinal barrier in patients with CD alters the gut–liver axis and contributes to the pathogenesis of hepatic disorders, such as MAFLD. For this reason, in recent years, growing interest has emerged in trying to outline the possible pathogenetic mechanisms underlying the association between CD and NAFLD in adults and children. 

## 5. Intestinal Barrier Dysfunction and the “Leaky Gut”

Since the interaction of gut and liver via enterohepatic circulation represents a potential link in the pathogenesis of several hepatic diseases, it has been supposed that the high pathological intestinal permeability in CD patients might represent the *primum movens* towards NAFLD development [36,37]. Not by chance, it has been assessed that patients with NAFLD have increased intestinal permeability and that steatosis is associated with small intestinal bacterial overgrowth (SIBO) [17,38]. Moreover, there is a correlation between the altered intestinal permeability, increased zonulin levels and the grade of steatosis. Therefore, lipotoxic molecules (e.g., cholesterol), LPS, and other substances can more easily pass through the intestinal barrier and activate the processes of apoptosis, oxidative stress, inflammation, and disruption of mitochondrial function [17].

In patients with active CD, the impairment of small intestinal mucosal barrier integrity is evidenced by the elevation of both the Cytokeratin 18 caspase-cleaved fragment, which is a marker of epithelial apoptosis, and the intestinal fatty acid-binding protein, which is marker of enterocyte damage [39]. In addition, a greater intestinal permeability has been reported in patients with active CD and elevation of serum transaminase than those with active CD and normal transaminase levels. Interestingly, a normalization of both gut permeability and transaminase levels after the beginning of a GFD have been documented [36,40].

Although for patients with CD, the altered intestinal barrier integrity is a consequence of the immune-mediated damage triggered by gluten intake, it is probable that gliadin *per se* interferes with zonulin, an important component of tight junctions (TJs), mediating its release and increasing intestinal permeability [26,41]. Moreover, circulating zonulin binds and activates CXCR3 thus inducing an increased production and release of gliadin mediated MyD88, which further contributes to TJs opening. CXCR3 is over-expressed in patients with CD both on immune cells and hepatic parenchymal cells. The role of these receptors in the healthy liver is not understood but, during injury, it promotes cellular survival, activation, proliferation, apoptosis, and inflammatory cell infiltration together with fibrogenesis, angiogenesis, and expression of additional chemokines and growth factors [42]. Recently, using an integrative multi-organ platform including human liver (hepatocytes and Kupffer cells) and intestinal (enterocytes, goblet cells, and dendritic cells) models, an overproduction of CXCR3 ligands promoted by inflammatory molecules deriving from the gut–liver axis has been documented [43].

To better understand the direct role of gluten in the disruption on intestinal barrier integrity, its effect in healthy models has been studied. Particularly, studies in no-CD mice exposed to gluten have evidenced an increased intestinal permeability in vitro (on Caco-2 epithelial monolayers) and an activation of the arginase metabolic pathway in monocytes/macrophages [44]. Therefore, the exacerbation of those mechanisms in CD brings the innate immune response activation and the releasing of inflammatory cytokines and nitric oxide (NO) by the activation of inducible NO synthase [45]. However, it is still unclear how those mechanisms really take part in the pathogenesis of NAFLD in patients with CD since they are also observed in healthy people [46].

Several studies demonstrated a link between the alteration of the intestinal permeability (the so-called “Leaky Gut”) and liver damage [38,47,48]. Physiologically, a small number of bacteria and their components overcome the intestinal barrier reaching firstly the mesenteric lymph nodes (MLN), where microbial killing occurs, and then the liver. In healthy individuals, when the microorganisms and their components escape the MLN surveillance, the liver acts as a second barrier against microbial agents. Particularly, Kuppfer cells expressed in hepatic sinusoid play a role in sentinels, capturing cellular residues and microorganisms from the blood circulation recognizing microbial- and pathogen-associated molecular patterns (MAMPs and PAMPs) [49,50]. PAMPs are small molecules including LPS, microbial DNA, peptidoglycans, and lipopeptides able to activate the local immune system cells and to determine an inflammatory state. Under the stimulus of interleukin-1 (IL-1) and interleukin-6 (IL-6), the liver produces the lipopolysaccharide binding protein (LBP), an acute phase protein able to bind LPS. LPS-LBP complex itself activates Toll-like receptor 4 (TLR4) and its co-receptor CD14, expressed on hepatic resident myeloid cells promoting their action [51,52,53]. The TLR4-LBP-LPS complex determines the activation of the NF-kB pathway and the expression of chemokines, vasoactive factors, and inflammatory cytokines such as TNF-α, IL-1β, IL-6, IL-12 and IL-18. This inflammatory storm induces the recruitment to the liver of systemic polymorphonucleates, monocytes, and CD4+ T cells, enhancing inflammation and inducing hepatocyte apoptosis and necrosis. These cells are also responsible for the expression of matrix metalloproteinases (MMPs) whose over-expression determines the destruction of hepatic tissue. Furthermore, both cell necrosis and cytokine storm stimulate the hepatic stellate cell (HSC) proliferation. The HSCs and the production of transforming growth factor-β (TGFβ) contribute to the development of hepatic fibrosis. This pathway is fundamental to ensure liver integrity. In fact, three studies conducted on TLR4 mutant mice highlighted the key role of TLR4 in the pathogenesis of liver diseases. They showed that the destruction of LPS-TLR4 signaling complex makes mice resistant to liver damage and fibrosis and protected against diet-induced overweight and insulin resistance (IR) [54,55]. Oxidative stress also plays a key role in the pathogenesis of liver diseases. In fact, reactive oxygen species (ROS) can affect both liver and gut. Several studies demonstrated that diet, infections, primary inflammatory diseases, and drugs may alter the redox state in the gut, resulting in greater intestinal permeability. The activation of TLR in the liver is responsible for the production of ROS by the Kupffer cells, causing the activation and proliferation of HSC [56]. In turn, Kupffer cells exposed to ROS produce cytokines and chemokines causing a vicious circle that determines ever higher levels of inflammation and cellular necrosis.

## 6. Gut Microbiome

Intestinal dysbiosis and alteration in the pathogenic/non-pathogenic bacterial species ratio are some of the conditions most frequently documented in subjects with CD. Although no clear and peculiar patterns of the celiac microbiota have been found, some studies have shown a reduction in protective and anti-inflammatory bacteria such as *Bifidobacterium* and *Lactobacillus* [37,57]. It is not yet clear if dysbiosis affects the intestinal permeability and TJs. However, alteration in microbiota composition contributes to intestinal inflammation [58] and to bacterial translocation and thus inflammatory activation of extraintestinal organs, including the liver. Particularly, alterations in the microbiota composition have been documented in the pathogenesis and maintenance of many liver diseases, including NAFLD [59,60]. It is currently unclear whether the type of diet affects the signature of microbiome. Studies conducted on mice fed with high-fat diets or fiber-deprived diets have shown an increased bacterial penetrability, reduced thickness of the intestinal barrier, redistribution of TJs of the epithelial barrier, and gut inflammation [61,62]. However, fecal microbial transplantation from mice fed a high-fat diet to mice fed a standard diet resulted in intestinal barrier damage. Collectively, these results indicate that it is not the nutritional regimen, but its consequences on the microbiota composition that disrupts the gut epithelium [63]. It seems that the altered microbiota acquires the ability to cross the epithelium, but it is not clear whether this is due to a greater invasiveness of pathobionts or due to a downregulation of TJs proteins. Certainly, dysbiosis, such as in untreated patients with CD, is responsible for a subclinical inflammation characterized by reduced lamina propria Treg cells, increased IFN-gamma-producing Th1, and CD8+ T cells, and increased IL-17-producing gamma-delta T cells [61]. Probably, this inflammatory state of the intestinal mucosa contributes to the increased permeability of the gut barrier.

Intestinal bacteria composition is also correlated with obesity and other metabolic diseases. Particularly, Turnbaugh et al. [64] suggested that obesity in mouse models may determine a significant decrease in the level of diversity in the species of gut microbiota. The authors suggested an analogy between the gut microbiota of obese mice and obese humans, assuming that even in patients with obesity it is possible to observe reduced diversity of the microbial community and abnormal energy input.

In conclusion, it is not clear when and how the differences in composition of the gut microbiota in patients with CD can cause metabolic alterations, such as NAFLD. However, it can be hypothesized that intestinal dysbiosis may determine a lower robustness of the intestinal barrier and an easier passage of endotoxins and xenobiotics into the systemic circle. From this point of view, the restoration of gut microbiota and integration with commensal bacteria could also be a therapeutic approach in the management of CD and NAFLD. Further studies are needed to understand how and when to intervene to restore a healthy composition of microbiome.

## 7. Bacterial Products and Metabolites

Most of the connections between the host and the microbiome are mediated by soluble factors, comprising bacterial products and metabolites, known as postbiotics [65,66]. Postbiotics include acetate, propionate, and butyrate, are overall defined short chain fatty liver acids (SCFAs), which are saturated fatty acids with one to six atoms of carbons [67,68] produced through microbiota bacterial fermentation of indigestible carbohydrates (e.g., dietary fiber). Several studies have shown an alteration in the production of SCFAs in some human intestinal diseases, such as IBD [67], irritable bowel syndrome [69], diarrhea [70], and cancer [71], raising the idea to use them as potential diagnostic biomarkers [72,73].

Although there is no available evidence about the association of SCFAs and CD, different associations between SCFA levels and gut permeability have supposed a potential interaction between them. Notably, it has been observed that SCFAs preserve the functionality of the intestinal barrier and counteract the onset of inflammatory reactions thanks to the regulation in the transcription of TJs proteins, in particular claudin-1 [73,74]. In addition, SCFAs support the proliferation and differentiation of colonocytes and protect the colon epithelium increasing the expression of mucin 2 and modulating both oxidative stress and the immune response [75]. SCFAs are able to modulate host immune responses modulating the number and functions of regulatory T cells (Treg) and stimulating the microbicidal activity of macrophages and production of antimicrobial peptides [76,77,78,79,80,81]. Soluble dietary fiber-rich diets are associated with higher SCFA production, which stimulate G-protein-receptors (GPR), the primary receptor for SCFA. GPCR inhibits NF-kB activation in immune cells and intestinal epithelial cells [82,83]. However, excessive levels of SCFA, and in particular higher levels of butyrate, have paradoxical effects, leading to the destruction of the intestinal barrier and hindering the assembly of TJs [84,85]. In addition, it seems that overproduction of SCFAs may determine the induction of nutrient transporters such as the glucose transporter 2 (GLUT2) in enterocytes, which increase the intestinal absorption of glucose and contribute to the development and maintenance of obesity [86,87,88,89]. Furthermore, SCFAs affect fat accumulation in liver through the regulation of insulin sensitivity via GPR43, a receptor that influences the inflammatory response with the regulation of inflammasome [90,91,92]. It is not clear whether the altered intestinal microflora in patients with CD could also lead to alterations in the production of SCFAs and whether this could also play a role in the pathogenesis of NAFLD. However, a study conducted by Tjellström et al. [93] demonstrated that children with CD at diagnosis and children with CD on a GFD for less than 1 year have significantly higher acetic acid, i-butyric acid, i-valeric acid, total SCFA, and fermentation index than healthy controls [93,94]. The authors speculated that in untreated patients with CD, higher levels of SCFAs are linked with altered digestion and absorption of carbohydrates and proteins in the small intestine, which is the major location of enteropathy in CD. In addition, it can be hypothesized that the dysbiosis in the small intestine, or a rapid passage through the small intestine, or decreased metabolic activity due to proximal small intestinal enteropathy, and malabsorption in patients with untreated CD contribute to an overload of undigested nutrients in the distal small intestine and colon [93]. Furthermore, it has been observed that children with CD on a GFD for more than 1 year show normalized intestinal microflora function with no differences from the controls in the SCFA and fermentation index [94]. There are currently no studies on how SCFAs can contribute to the pathogenesis of NAFLD in patients with CD. Certainly, elevation of SCFAs has been documented in adult and pediatric patients with NAFLD. Specifically, Bacteroidetes, which produce more SCFAs than other phyla [95], are significantly elevated in obese adults and children with NASH compared to healthy controls [96,97]. These data are also confirmed by Rau et al. who showed higher fecal SCFA concentrations in NAFLD patients than healthy controls, and higher SCFA concentrations are also associated with progression from NAFLD to NASH [98]. In patients with CD, it can be hypothesized that the excessive amount of SCFAs in the colon may contribute to the alteration of the gut barrier and to the disassembly of TJs. Elevated levels of SCFAs could also play a role in the induction of nutrient transporters, such as GLUT2, favoring intestinal glucose absorption and contributing to the onset of obesity and therefore MAFLD. Further studies aiming to highlight the effects of increased microbiota production of SCFAs are needed.

## 8. Malnutrition and Compensatory Theory

Semeraro first hypothesized that the intestinal atrophy in patients with CD could be compensated by enhanced absorption in the distal bowel segments. Not casually, the fat absorption coefficient could be maintained in a patient with partial gut atrophy [99] Similarly, in patients with CD, the loss of normal intestinal function in the atrophic areas is responsible for the increased absorption of the functionally preserved intestinal tract. However, the excessive absorption of nutrients as a compensatory mechanism might lead to a positive energy intake, thus increasing the risk of overweight/obesity. This compensatory hypothesis appears to be supported by some of the first published cases of adolescents affected by CD who continued to present with overweight or obesity regardless of persistent villous atrophy on intestinal biopsies [100,101,102,103,104]. It seems that there is a positive correlation between the compensatory surface area of the small intestine and patient age. For that reason, older children, adolescents, and adults often present with atypical symptoms than younger who classically present symptoms of malabsorption at diagnosis. Beyond the increased risk of overweight and obesity, malnutrition leads itself to mitochondrial dysfunction in hepatic cells and to a reduction in mitochondrial fatty acid β-oxidation, responsible for the increased hepatic fat deposition [105]. A GFD usually leads to the resolution of liver steatosis in patients with CD [106]. This phenomenon might be partly explained by the improved nutritional status. Furthermore, it seems that a GFD may contribute to the restoration of innate immune liver function via harmonization of the gut–liver axis [107].

## 9. Bile Acids (BAs) as Mediator of Hepatic Damage in CD Patients

Recently, scientific interest has been focused on the potential role of bile acids (BAs) in the pathogenesis of many diseases, including NAFLD. In this regard, BAs have been identified as interesting mediators of communication between gut and liver with a probable role in the pathogenesis of NAFLD. Primary BAs (cholic acid (CA) and chenodeoxycholic acid (CDCA)) are produced from cholesterol in hepatocytes through the reaction catalyzed by cholesterol-7-alpha-hydroxylase (CYP7A1). This enzyme acts as a controller of BAs synthesis, since its expression is regulated by negative feedback based on CA levels through the activation of farnesoid X receptor (FXR). The jointed activation of FXR and its hepatic target gene NR0B2 controls the production of FGF-19, which repress the CYP7A1 gene expression. After the conjugation to glycine and taurine, primary BAs are secreted into the biliary canaliculi with the bile that is partially stored in the gallbladder. In contrast, under the stimulus of cholecystokinin (CCK) released after a meal, they are injected in the intestinal tract where they facilitate the digestion and absorption of fatty acid, and lipophilic vitamins (A, D, E, and K). In the intestine, BAs are turned into secondary bile acids (deoxycholic acid and lithocholic acid) by local bacterial flora, thus reabsorbed in the ileum to return to the liver through portal circulation. Considering the tight association between liver and gut, several authors have supposed that alterations in BAs metabolism and/or gut–liver interaction has a pivotal role in the pathogenesis of hepatic steatosis [108,109,110]. In this regard, a recent case–control study conducted on 20 adult CD patients on a gluten-free diet in clinical remission matched with 20 healthy controls, observed an increase in the apoptosis marker M30 and higher severity of steatosis in the former than the latter. Particularly, authors observed that low levels of Fibroblast Growth Factor 19 (FGF-19), normally repressed in CD patients on a GFD, and anti-tTG positivity are associated with a higher prevalence of hepatic steatosis, supposing a direct role of BAs in hepatic steatosis pathogenesis [111]. A study conducted in a pediatric population also evaluated the different preprandial and postprandial BA profiles by varying intestinal damage [112]. The authors observed that about 45% of symptomatic celiac patients at the diagnosis with alterations in standard liver function tests (ALT, AST, and/or GGT) have higher levels of fasting BAs than patients with normal tests. No differences have been reported in postprandial BAs patterns. These results might imply that the liver play a primary role in determining BAs levels in this group of patients. However, also gut factors regulates BA pools. Indeed, the delayed BA postprandial pick in patients with CD might be attributable to a delayed emptying of the gallbladder because of the reduced production of intestinal CCK or for a natural resistance to the action of CCK. As evidence of this, untreated patients with villus atrophy or patients who have undergone a gluten challenge have a similar BA pattern suggesting that the intestinal damage influences BA pool [113,114,115,116]. Notably, different conjugate BA absorption patterns have been described in pediatric patients with CD. This can be partly due to the different intestinal damage, but also to the different gut microbial population that interfere with deconjugation. However, the persistence of fasting and postprandial BA pool trends also in pediatric-treated CD patients with histological remission suggests that there is probably an alteration in BA metabolism in those subjects, partly due to a persistent large BA pool and partly due to a gut/liver influence [112]. Particularly, an improvement in gallbladder function might increase the afflux of BAs to the liver increasing their pool. Further studies are necessary to better investigate this strong relationship to obtain a pathogenetic model.

## 10. The Role of Tissue Transglutaminase (TTG2) Antibodies and Vitamin D

TTG2 antibodies are the main protagonists of the pathogenesis of CD being directly responsible for the reversible intestinal damage. Considering the high specificity of TTG2 antibody levels for CD and that it is not rare to find elevate levels of circulating transaminases in patients with CD, it has been supposed that TTG2 antibodies might have a role in the pathogenesis of CD-associated liver damage [37]. To support this hypothesis, it has been reported that other small intestinal diseases associated with dysbiosis and an increased inflammatory mucosal response such as sprue or diarrhea predominant irritable bowel syndrome do not present a similar association with hypertransaminasemia, thus highlighting a potential role of TTG2 [117]. 

TTG2 is an enzyme involved in many biological processes, likely including wound healing, tissue repair, fibrogenesis, apoptosis, inflammation, and cell-cycle control. Particularly, it mediates cell–extracellular matrix interactions and contributes to the remodeling and stabilization of the extracellular matrix through transforming growth factor (TGF)-β [118,119,120,121]. There is evidence that TTG2 is highly expressed in the liver of patients affected with chronic liver disease where it acts as modulator of the process of fibrogenesis and inflammation through a mechanism that is not completely understood. Specifically, TTG2 is overexpressed in patients affected simultaneously with liver diseases (toxic hepatitis, primary sclerosing cholangitis, or autoimmune hepatitis type I) and active CD, offering a potential protective action [119,120]. The elevated TTG2 antibodies titer in patients with CD reduce the availability of TTG2 with a consequent reduction in its cellular and extracellular benefits [121]. 

It has been suggested that vitamin D deficiency, a usual finding in celiac patients [122], might also contribute to a generation of an inflammatory state responsible for successive liver damage. Particularly, vitamin D inhibits the maturation of dendritic cells, modulates the responsiveness of macrophages to LPS and other MAMPs, and facilitates the homing of activated T cells [123].

## 11. Genetic Contribution

The only exposition to a dysmetabolic environment does not explain the variability in the MAFLD development existing between people exposed to the same environmental factors. Therefore, it has been suggested that genetic factors might be implicated in the development and progression of liver damage in patients with NAFLD/MAFLD [124,125]. Thanks to the genome wide-association study (GWAS) different genetic variants associated with the NAFLD phenotype have been identified [126]. Among them, the most common encode for proteins involved in lipid metabolism and inflammation [127,128]. Particularly, the rs738409 C>G single nucleotide polymorphism of PNPLA3 is the first best studied factor of susceptibility [127]. The gene codifies for a protein involved both in lipid remodeling of hepatic droplets and modulating the activation of inflammatory cascade in the liver. As a consequence of the replacement of cytosine with guanidine, the protein loses its functionality predisposing to fatty liver and subsequent events leading towards NASH. The association between PNPLA3 polymorphisms, hepatic steatosis, obesity, and MetS are still debated [129,130,131,132]. Mangge et al. [130] observed a correlation between the PNPLA3 rs738409 polymorphism and hypertransaminasemia in young people, especially in obese subjects with MetS. Furthermore, Shen et al. [131] described the PNPLA3 rs738409 genotype as the greatest predictor of hepatic steatosis in the absence of MetS. Conversely, Del Ben et al. [132] reported that the PNPLA3 I148M gene variant was associated with a lower prevalence of MetS and reduced cardio-metabolic risk in adult subjects with hepatic steatosis. Recent studies carried out on an adult population with CD have demonstrated that PNPLA3 I148M gene variant is associated with the development and severity of hepatic steatosis, but not related to metabolic disorders [129,133]. Although no other polymorphisms have been associated with NAFLD or MAFLD, this first evidence might offer a cue to further investigate the possible pathogenetic aspects.

## 12. GFD and MAFLD

Nowadays, a GFD is the only accepted strategy to safely treat patients with celiac disease. However, it is not exempt from adverse effects [6,134]. The last scientific evidence reports that its composition in terms of macronutrients might predispose individuals to an unbalanced diet and lead to weight gain. In this context, a GFD might play a pivotal role in the evolution towards the clinical and laboratory features of obesity and MetS in patients with CD. The reason lies partly in changes in eating regimens. In fact, patients on a GFD, in order to supply the reduced sources of allowed foods, consume higher amounts of products rich in sugars, proteins, saturated fats, and complex carbohydrates, and have an increased intake of daily calories. In addition, gluten-free products are featured by a higher glycemic index than gluten-contained foods, predisposing individuals to the development of insulin resistance (IR). Therefore, a GFD may have a negative impact on cardio-metabolic risk factors such as obesity, serum lipid concentrations, IR, MetS, and atherosclerosis, driving towards NAFLD/MAFLD development [135]. However, to date, this concept still remains under discussion. Studies in adults have suggested that GFDs have a beneficial effect on cardiovascular profile consequently to its anti-atherosclerotic effects [136,137], whereas others have shown atherogenic effects of a GFD [7]. Instead, in children, very few studies are available on this topic [135]. A recent retrospective study conducted by Rispo et al. [133] on a population of 221 newly diagnosed adult patients with CD matched the prevalence of NAFLD and MAFLD at the time of diagnosis and after two years of follow-up. They observed a higher prevalence of NAFLD vs. MAFLD at diagnosis (29.4% and 14.5%, respectively) and for both, they documented an increased trend at the end of the defined period of follow-up (46.6% and 32.6%, respectively). As expected, the only difference between MAFLD and NAFLD at diagnosis was the higher rate of IR among MAFLD patients (75% vs. 33.8%). Nonetheless, after 2 years of follow-up, higher levels of transaminases, LDL-C, insulin serum, HOMA index, weight, BMI, waist circumference, and IR were observed in MAFLD than NAFLD patients. However, since the majority of patients with NAFLD (81.8%) “progressed” to MAFLD after 2 years of a GFD, questions have been raised about the possibility to consider MAFLD as a negative prognostic factor. Considering the strong association existing between CD and metabolic alterations, in the following paragraphs we discuss the current knowledges about the relationship between CD and Mets.

### 12.1. Obesity

The weight gain in patients with CD may be linked to the global trend toward overweight and obesity in pediatric age [138]. The proportion of children with CD affected by overweight and obesity at diagnosis is between 8.8% and 20.8% and between 0% and 6%, respectively [6]. Regarding the effects of a GFD on obesity, the scientific literature provides contrasting data. There is evidence that good compliance with a GFD is associated with a positive effect on anthropometric parameters, including reduction in excessive corporeal fat accumulation [139], normalization of BMI in both previously underweight and overweight subjects [140], and acceleration in linear growth [141]. Particularly, Brambilla et al. evaluated the BMI changes at the time of CD diagnosis until the latest clinical assessment in a relatively large group (150 subjects) of children with CD on a GFD (median time, 4.4 years) compared to 288 age and sex-matched healthy controls. Authors showed that the frequencies of overweight and obesity in children with CD at diagnosis and during a GFD were significantly lower than those reported in healthy controls [142]. Even though the frequency of being overweight and obese in the CD group increased on the GFD, it remained lower than observed in the general population [143]. The study by Reilly et al. [134] involving 142 pediatric patients with CD showed that 19% of subjects at diagnosis presented an elevated BMI (12.6% were overweight and 6% were obese), while 74.5% had a normal BMI. Seventy-five percent of children with high BMI at diagnosis had a significant decrease in their BMI z-scores on a strict GFD. In particular, 44% of them normalized their BMI z-scores. The authors concluded that GFD may have a favorable effect upon the BMI of overweight and obese youths with CD. On the other hand, there is some evidence that a GFD may predispose individuals to the development of overweight and obesity [135]. The improved intestinal absorption subsequent to the progressive resolution of intestinal atrophy might represent a key element for the successive excessive fat accumulation. However, the high content of lipids and proteins of gluten-free foods in association with the tendency to consume foods with elevated caloric fat and protein content to contrast the GFD unpalatability represents the most important factors predisposing to fat accumulation [144]. There are multiple reports on the association of a GFD and obesity in youths with CD, with conflicting results. Mariani et al. [144] first reported a higher rate of overweight and obesity in adolescents with CD on a GFD in comparison with CD subjects on a gluten-containing diet and healthy controls (72% vs. 51% and 47%). Valletta et al. [6] found that the prevalence of overweight and obesity was 11% and 3%, respectively, at diagnosis in 149 youths with CD. After at least 12 months of a GFD, there was a significant increase in the body mass index (BMI) z-score, and the percentage of overweight children almost doubled (11% vs. 21%). The proportion of obese patients remained the same (3% vs. 4%). In a recent cross-sectional multicenter study, Norsa et al. [7] evaluated 114 CD children with negative CD serology after at least 1 year of a GFD. They found that 9.6% of youths were underweight, 76.3% had normal weight, and 8.8% were overweight, whereas 5.3% were obese. Both prevalence of overweight and obesity rose up to 11.4% and 8%, respectively, after GFD introduction. This conflicting data may partly be caused by differences in the timing of anthropometric assessment. In fact, children with CD may show excessive weight gain at the start of a GFD and only thereafter start to normalize their weight [143]. Even though the effect of a GFD on body weight and BMI remains a controversial issue, it remains fundamental for pediatricians to be aware of the possible nutritional consequences of a GFD for which early recognition can be crucial in the prevention of obesity-related complications.

### 12.2. IR and Glucose Metabolism Alterations 

There are only a few studies on IR in both adults and youth with CD on GFDs. In a retrospective study evaluating the effects of 1–5 years of a GFD on markers of cardiovascular risk in a large cohort of adult patients, Zanini et al. [136] found that IR remained constant from baseline to follow-up. Of note, absolute levels of serum glucose rose from 87.9 mg/dL at baseline to 89.7 mg/dL during a GFD, reaching a statistical significance only in females. In a sample of Italian and Israeli patients with CD on a GFD, Norsa et al. [7] found that 3.5% of 114 children on a GFD had IR. Because of the lack of data on insulin values before CD diagnosis, the authors were not able to determine whether IR could indeed be attributed to the start of a GFD. Although the association between GFD and IR is not yet clear, the increased rate of obesity represents a key element in the establishment of a state of IR. Furthermore, a strong relationship between Hepatic Fat Fraction (HFF) and IR has been described. Therefore, IR cannot be extraneous to the pathogenesis of MAFLD also in patients with CD [145].

Alteration in blood glucose levels also appears significantly different in patients with CD at diagnosis and after 1 year of a GFD, with an approximate 4.5-fold increase in risk for onset of hyperglycemia [146]. Certainly, elevated fasting plasma glucose levels are an expression of IR, directly related to MetS together with central obesity. IR and obesity are two entities strongly associated with each other. Particularly, tumor necrosis factor-a (TNF-a), whose expression has been demonstrated to be increased in human obesity, contributes to generate IR by inhibiting tyrosine kinase activity at the insulin receptor. Therefore, the BMI increase secondary to a GFD might related to the higher prevalence of IR in patients with CD at follow-up [147,148,149]. Another hypothesis about the pathogenesis of IR is directly related to CD. Indeed, TTG2 mediates pro-inflammatory action in patients with CD downregulating the peroxisome proliferator-activated receptor c (PPARG), whose activation is implicated in type 2 diabetes susceptibility [150,151]. So, it is possible that by reducing inflammation, the GFD might influence this pathway. However, this hypothesis needs to be validated by further studies.

### 12.3. Serum Lipids

The consequences of a GFD on lipid profile in childhood have been analyzed in a few studies, most of them regarding patients at diagnosis and shortly after the start of a GFD. Rosenthal et al. [152] compared plasma lipids and lipoprotein pattern between 12 untreated CD children and 10 control subjects. They found an altered lipid profile characterized by decreased serum triglyceride (TGs) levels, very low-density lipoprotein (VLDL) and apolipoprotein A-I and increased low-density lipoprotein cholesterol (LDL-C) in children with CD, because of fat malabsorption. On a GFD, the lipid profile reverted to normal as consequence of improvement in mucosal damage caused by gluten withdraw. Ciampolini et al. [153] investigated the plasma lipid levels in children with CD (45 under and 49 over 3 years of age, at diagnosis) on GFDs, and in comparison, with children affected by irritable bowel syndrome matched for body size, gender, and age. They found that untreated children with CD had significantly lower total and high-density lipoprotein cholesterol (HDL-C) levels and significantly higher TG concentrations than controls in both age groups. A period of GFD increased HDL-C in both age groups, while total cholesterol, LDL-C, and TGs levels decreased only in the younger age group. Pillan et al. [154] compared serum lipids and lipoprotein (a) concentration in 17 adult and pediatric patients with CD at diagnosis and after 3 months of GFD. They found that mean total cholesterol and LDL-C did not significantly change, while mean HDL-C significantly rose and mean triglyceride levels significantly decreased after the diet, suggesting that GFD may positively influence lipid profile. Recently, in a cross-sectional prospective study, Forchielli et al. [155] analyzed the impact of long term GFD on serum lipid concentrations in children with CD comparing them with the general pediatric population. They studied 235 children and adolescents with CD. In this study, the lipid profile was available only after diagnosis (group 1) in 205 patients, whereas in 30 patients it was available both before and after GFD (group 2). The authors found that in group 1, total cholesterol (TC), triglycerides, and HDL-C concentrations were significantly elevated in girls in comparison with boys. Furthermore, compared with the general pediatric population, group 1 girls had higher TC, TGs, HDL-C and LDL-C, while group 1 boys had lower TC, TG and LDL-C, and higher HDL-C. In group 2, TC did not change over time, TG diminished, and HDL-C increased. These changes in the liver and circulating profiles combined with IR well explain the imbalance existing within circulating FFAs and DNL. Physiologically, the elimination of excess TGs produced in the liver is mediated by both *β*-oxidation and elimination as VLDL. In subject with NAFLD there is a reduction in the secretion of hepatic VLDL into blood stream. As a consequence, the excess of DNL and VLDL storage contribute to fat accumulation [156]. 

### 12.4. Metabolic Syndrome (MetS)

It is no uncommon to find children and adolescents with CD featured by high waist circumference, hypertension, hypertriglyceridemia, hypercholesterolemia, and diabetes mellitus, classically featuring MetS. In this context, liver steatosis seems to appear as the hepatic manifestation of the general metabolic dysregulation and the new definition of MAFLD well describe the effect of this dysmetabolic state on liver anatomy and function. Data regarding the prevalence of MetS in patients with CD on a free diet or on a GFD are still scarce and limited to adults with CD. In an observational prospective study involving 98 adult patients with CD, Tortora et al. [146] found that 2% of them fulfilled the diagnostic criteria for MetS at diagnosis while 29.5% fulfilled the criteria after one year of a GFD. Among the MetS components, there was a significant increase in BMI, waist circumference, blood pressure (BP) and blood glucose levels, whereas no significant difference was detected in the lipid profile from baseline to 12 months after the introduction of a GFD. 

## 13. Final Considerations and Conclusions

There is wide evidence supporting the association between CD and NAFLD both in adults and in children. Therefore, these reports strongly suggest that the association cannot be considered a casualty. Unfortunately, the exact pathogenetic mechanisms linking these two diseases are not yet known. The most reliable explanation supports the idea that metabolic imbalance acts as a trigger for the hepatic fat accumulation and inflammation. In support of this suggestion, all patients with CD who start a GFD are subjected to weight gain, increases in BMI z-score, circulating TGs and TC levels as well as to an increased risk to develop NAFLD. It remains to be clarified if these metabolic alterations are favored by the increased intestinal absorption secondary to the regression of duodenal mucosal atrophy or by the elevated consumption of GFD, especially of industrial origin, rich in fats and simple carbohydrates. However, recent studies have observed that, despite a good adherence to a gluten-free diet, in the same patients, the increased gut permeability persists. Therefore, this condition might promote an increased afflux of toxic exogenous components of infectious and non-infectious origin able to activate intestinal and hepatic immune cells through TLR. Furthermore, changes in the microbiota population have been evaluated as possible factors implicated in the increased permeability of the intestinal barrier and in the direct activation of the inflammatory cascade. Taking into account this recent evidence, the actual scientific interest is addressed to better understand the functional connections existing between the liver and the gut. Especially, researchers are trying to investigate the so-called “gut–liver axis” also in patients with CD to obtain information helpful for the management of hepatic consequences. A pathogenetic link between increased gut permeability, microbiota, and diet can help to understand the role of the gut–liver axis in the development of NAFLD. However, other factors seem to play a role. Based on the available data, it can be concluded that the serological evaluations for CD with anti-tTG should be a part of the general workup in the asymptomatic patients with NAFLD when metabolic risk factors are not evident, and in the patients with steatohepatitis when other causes of the liver disease are excluded. Notwithstanding further information, it appears necessary to monitor liver function, body weight, and metabolic and nutritional profiles in all patients with a new diagnosis of CD.

## Data Availability

Not applicable.
